# P-364. Efficacy and Safety by Sex Assigned at Birth After Switch to Doravirine/Islatravir (100 mg/0.25 mg) Once Daily: Week 48 Results from Two Phase 3 Randomized, Active-Controlled Studies in Adults Living with HIV-1

**DOI:** 10.1093/ofid/ofaf695.582

**Published:** 2026-01-11

**Authors:** Princy N Kumar, Sharon Walmsley, Carolina E Chahin Anania, Eugenie Colin-Benoit, Alireza Farabi, Jaclyn Ann Bennet, Elizabeth Hellstrӧm, Alexandra Calmy, Cynthia C Brinson, Chloe Orkin, Anjana Grandhi, Monica Fuszard, Stephanie O Klopfer, Rima Lahoulou, Luisa M Stamm, Michelle C Fox, Jason Y Kim

**Affiliations:** Georgetown University Medical Center, Washington, DC, USA, Washington, DC; University Health Network, Toronto, Ontario, Canada; Hospital Hernán Henríquez Aravena, Temuco, Araucania, Chile; Department of Infectious Diseases, Inselspital, Bern University Hospital, University of Bern, Bern, Bern, Switzerland; Farabi Wellness Center, Las Vegas, Nevada; Clinical HIV Research Unit CHRU, Wits Health Consortium WHC, Health Science Research Office HSRO, Faculty of Health Sciences, University of Witwatersrand, Johannesburg, Gauteng, South Africa; Be Part Research PTY LTD, Paarl, Western Cape, South Africa; Division of Infectious Diseases, Geneva University Hospital, University of Geneva, Geneva, Geneve, Switzerland; Central Texas Clinical Research, Austin, TX, USA, Austin, Texas; Queen Mary University of London, london, England, United Kingdom; Merck & Co., Inc., Rahway, NewJersey; Merck & Co., Inc, Rahway, NewJersey; Merck & Co., Inc., Rahway, NewJersey; MSD, Puteaux, Haute-Normandie, France; Merck & Co., Inc., Rahway, NewJersey; Merck & Co., Inc., Rahway, NewJersey; Merck & Co., Inc., Rahway, NewJersey

## Abstract

**Background:**

In two Phase 3 studies, switching to doravirine/islatravir (DOR/ISL, 100 mg/0.25 mg), an investigational once-daily regimen for HIV treatment, was non-inferior for efficacy with a safety profile comparable to continuing baseline antiretroviral therapy (bART) or bictegravir/emtricitabine/tenofovir alafenamide (BIC/FTC/TAF) at Week 48 (W48). Demographic parameters might affect the efficacy or adverse event (AE) profile of an antiretroviral regimen. This subgroup analysis was performed to evaluate the efficacy and safety of DOR/ISL by sex assigned at birth.

**Figure:**
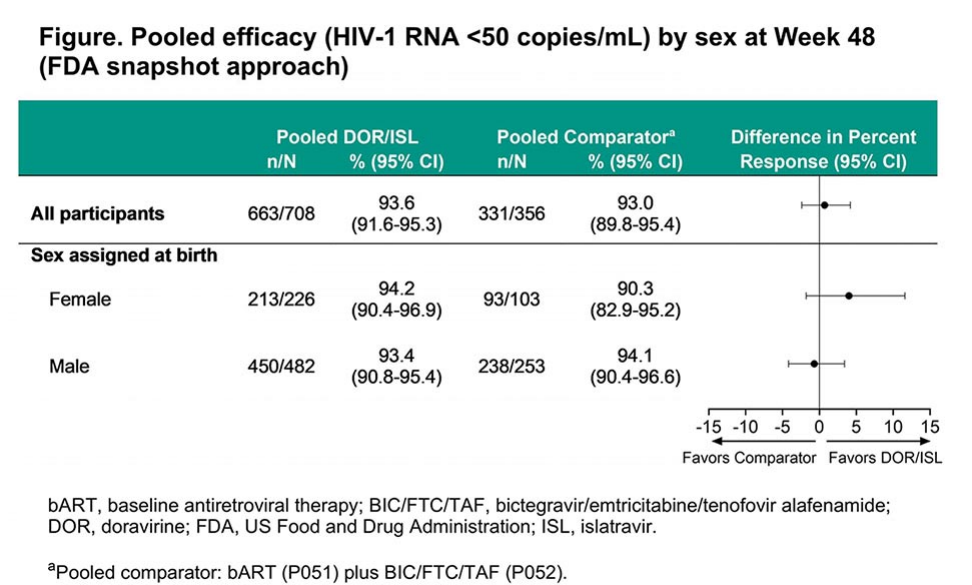

**Figure:**
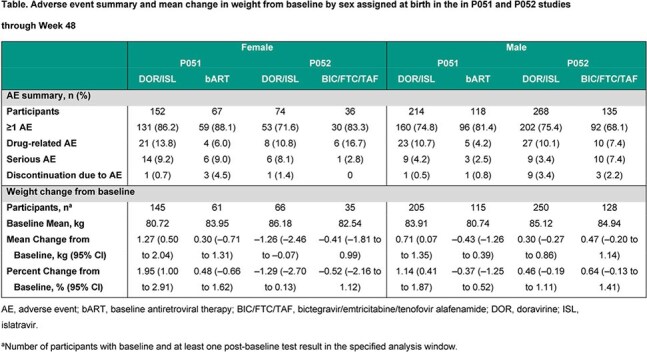

**Methods:**

Adults with HIV-1 RNA < 50 copies/mL receiving stable oral bART (MK-8591A-051 [P051]; NCT05631093) or BIC/FTC/TAF (MK-8591A-052 [P052]; NCT05630755) for ≥3 months were randomized (2:1) to switch to DOR/ISL (100 mg/0.25 mg), or to continue bART (P051) or BIC/FTC/TAF (P052). For P051+P052, efficacy results were pooled for both the DOR/ISL arms and the comparator arms (pooled comparator) and were summarized by sex assigned at birth (female or male) through W48. AEs and weight were reported by sex subgroup for each study.

**Results:**

Across both studies (P051+P052), 708 participants switched to DOR/ISL and 356 continued bART or BIC/FTC/TAF (pooled comparator); overall 30.9% were female. At W48, the proportion of participants with HIV-1 RNA < 50 copies/mL was similar between females and males in the pooled DOR/ISL group (94.2% vs 93.4%) and generally similar to the pooled comparator group (90.3% vs 94.1%; Figure). In open-label P051 in all treatment groups, females had consistently higher rates of AEs and drug-related AEs than males (Table). In double-blind P052, AEs and drug-related AEs were comparable for females and males in the DOR/ISL group. The rates of discontinuation due to AEs by sex were low and < 5% in all treatment groups. For females and males who switched to DOR/ISL, change in weight from baseline was minimal (mean percent change range in sex subgroups −1.29% to 1.95%; Table). There were no clinically meaningful differences in weight change between females and males.

**Conclusion:**

At W48, switching to DOR/ISL (100 mg/0.25 mg) demonstrated high efficacy and was generally well tolerated in females and males living with HIV-1.

**Disclosures:**

Princy N. Kumar, MD, Gilead Sciences, Inc (Foster City, CA, USA): Grant/Research Support|Gilead Sciences, Inc (Foster City, CA, USA): Medical writing support provided by Aspire Scientific (Bollington, UK)|Gilead Sciences, Inc (Foster City, CA, USA): Stocks/Bonds (Public Company) Sharon Walmsley, MD, Gilead Sciences: Advisor/Consultant|Gilead Sciences: Grant/Research Support|Gilead Sciences: CME Speaking Engagements|GSK: Advisor/Consultant|GSK: Grant/Research Support|GSK: CME Speaking Engagements|Janssen: Advisor/Consultant|Janssen: Grant/Research Support|Janssen: CME Speaking Engagements|Merck: Advisor/Consultant|Merck: Grant/Research Support|Merck: CME Speaking Engagements|ViiV Healthcare: Advisor/Consultant|ViiV Healthcare: Grant/Research Support|ViiV Healthcare: CME Speaking Engagements Alexandra Calmy, MD, PhD, Gilead Sciences: Grant/Research Support|MSD: Grant/Research Support|ViiV Healthcare: Grant/Research Support Cynthia C. Brinson, MD, Gilead Sciences, Inc.: Grant/Research Support|Gilead Sciences, Inc.: Honoraria|Gilead Sciences, Inc.: Medical writing funds|GSK: Grant/Research Support|ViiV: Grant/Research Support Chloe Orkin, MBChB, FRCP, MD, AstraZeneca: Grant/Research Support|Bavarian Nordic: Payment directly from commercial firms for lecture(s), including service on speakers’ bureaus; Travel Reimbursements|Gilead Sciences, Inc: Grant/Research Support|Gilead Sciences, Inc: Payment directly from commercial firms for lecture(s), including service on speakers’ bureaus; Travel reimbursement|GlaxoSmithKline,: Grant/Research Support|GlaxoSmithKline,: Payment directly from commercial firms for lecture(s), including service on speakers’ bureaus ; Travel Reimbursement|Janssen: Grant/Research Support|Janssen: Payment directly from commercial firms for lecture(s), including service on speakers’ bureaus|MSD Pty LTD: Grant/Research Support|MSD Pty LTD: Payment directly from commercial firms for lecture(s), including service on speakers’ bureaus|ViiV Healthcare: Grant/Research Support|ViiV Healthcare: Payment directly from commercial firms for lecture(s), including service on speakers’ bureaus ; Travel Reimbursements Anjana Grandhi, PhD, Merck & Co., Inc.: Employment|Merck & Co., Inc.: Stocks/Bonds (Public Company) Monica Fuszard, MS, Merck & Co.: Employment Stephanie O. Klopfer, PhD, Merck & Co., Inc: Employment|Merck & Co., Inc: Stocks/Bonds (Public Company) Rima Lahoulou, n/a, MSD: Employment|MSD: Stocks/Bonds (Private Company) Luisa M. Stamm, MD, PhD, Merck & Co., Inc.: Employment|Merck & Co., Inc.: Stocks/Bonds (Public Company) Michelle C. Fox, MD, Merck & Co., Inc.: Employment|Merck & Co., Inc.: Stocks/Bonds (Public Company) Jason Y. Kim, MD, MSCE, Merck & Co.: Employment|Merck & Co.: Stocks/Bonds (Public Company)

